# Diffuse Leptomeningeal Glioneuronal Tumour

**DOI:** 10.7759/cureus.38404

**Published:** 2023-05-01

**Authors:** Tom Saliba, Grammatina Boitsios

**Affiliations:** 1 Radiology, Hopital Universitaire Des Enfants Reine Fabiola, Brussels, BEL; 2 Radiology, Université Libre de Bruxelles, Brussels, BEL; 3 Pediatric Neuroradiologist, Hopital Universitaire Des Enfants Reine Fabiola, Brussels, BEL

**Keywords:** brain tumors cns tumors, brain spine tumors, diffuse leptomeningeal glioneuronal tumor, diffuse glioneural leptomeningeal tumor, dglnt

## Abstract

Diffuse leptomeningeal glioneuronal tumours (DL-GNT) are rare, with an unknown incidence but fewer than 100 cases reported since 2012. The clinical presentation is non-specific, ranging from abdominal to neurological symptoms. Presently, definitive radiological criteria aren’t established, but some features, such as nodules, characteristic extension patterns and post-contrast leptomeningeal enhancement, are found to be prominent. We present the case of a 14-year-old male with an advanced case of DL-GNT, with MRI showing all the features of what is currently thought to be the typical radiological presentation. The patient is currently undergoing treatment but remains severely handicapped by the disease.

## Introduction

Diffuse leptomeningeal glioneuronal tumours (DL-GNT) are rare, with the true incidence being unknown due to the limited number of cases [1,2]. The tumours are mainly found in children, with the median age at diagnosis being five years old, though rare cases have been found in adults [1-4]. According to some authors, males are more often affected [1]. Owing to its rarity, the aetiology, natural progression and exact prognosis remain unknown [2]. Furthermore, there currently exists no WHO grade for this tumour [1,2]. The tumour was only inducted into the WHO classification in 2016 [1,2]. We present the case of a particularly advanced case of DL-GNT with prominent radiological features in a 14-year-old boy who had been diagnosed 10 years prior.

## Case presentation

**Figure 1:**
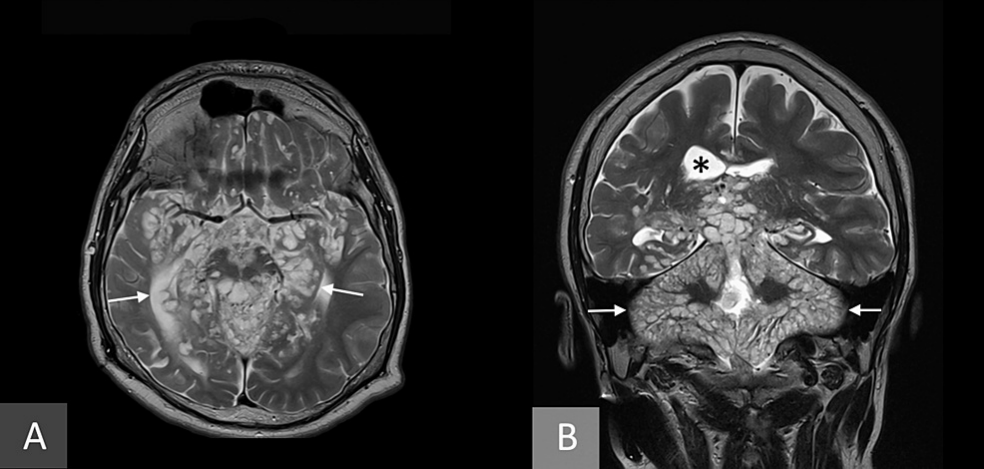
Axial (Panel A) and coronal (Panel B) T2-weighted MRI, showing extensive subpial cysts (arrows) alongside the hydrocephalus (star)

A 14-year-old male presented for a follow-up MRI of a DL-GNT, diagnosed 10 years prior. When the disease first revealed itself 10 years prior to the current exam, the patient had initially presented with neurological symptoms, with a trembling left upper eyelid and left side of the lip accompanied by acrocyanosis, prompting a hospital admission. The patient had a generalised convulsive episode after exiting the ambulance, prompting an MRI which revealed amygdala ptosis with tri-ventricular hydrocephalus and infra-tentorial arachnoiditis. This was followed by a surgical decompression to relieve the pressure, accompanied by a biopsy. The histopathology showed hyperplasic endothelium in the vessel linings of the meninges but no pseudo-nodular aspect. The tumoural proliferation within the brain parenchyma was nodular and infiltrative with the destruction of the granular layer. There was no necrosis, granuloma or multinucleated giant cells. The immune markings were negative for anti-Neu-N (Chemicon, clone A60) and EMA (Leica, clone GP1.4). The immune markings were positive for anti-NSE (Dako, clone BBS/NC/VI-H142), beta-3 tubulin (R&D, clone TUJ-1) and NFP (Dako, clone 2F11), confirming the infiltrative nature of the tumour. The tumour cells were positive Synaptophysin in the parenchyma but only weakly so in the meninges. The anti-GFAP (Dako, clone 6F2) markings favour oligodendroglial cell types within the parenchyma but are negative in the meninges. The anti-OLIG2 (Chemicon polyclonal) markings were diffusely positive. The anti-P53 (Dako, clone DO-7) was expressed in over 10% of tumoural cells. The KI67 (Dako, clone MIB-1) proliferation index was globally low at around 1%, with some areas being superior to 3%. There were few areas of mitotic activity. The biopsy was compatible with a diagnosis of DL-GNT. The patient began chemotherapy treatment according to the International Society of Paediatric Oncology-Low Grade Glioma subcommittee 2004 protocol, with the addition of bevacizumab. Two years prior to the current admission, an anatomopathological sample had been taken. A search for 10 known fusion patterns of the BRAF and KIAA1549 genes was conducted using a BRAF-KIAA1549 fusion panel (Ion Gene Studio S5, Ion Torrent with Kit Ion AmpliSeqTM RNA BRAF-KIAA1549 fusion panel) showing a KIAA1549(14)-BRAF(9) (COSF483) fusion, characteristic of pilocytic astrocytomas but also known to be found in glioneuronal tumours with diffuse leptomeningeal extension. A search for 14 more mutations known to occur in gliomas and 1p19q co-deletions (Ion Gene Studio S5, Ion Torrent with Kit Ion AmpliSeqTM) showed no mutations in the ACVRI, ATRX, BRAF, CDKN2A, EGFR, H3F3A, HISTH3B, HIST1H3C, IDH1, IDH2, PDGFRA, PTEN, TERT, TP53 genes or 1p19q co-deletions, though this result was to be taken with caution due to the low number of tumoural cells in the sample. Other genetic tests on the sample tissue were negative. During this routine follow-up, an MRI was performed, showing extensive subpial cysts, hyperintense in T2-weighted imaging (T2WI), in the brain. These were very extensive, centred around the midline in the supratentorial space, and involved the near totality of the infratentorial brain (Figure 1A, B).

**Figure 2:**
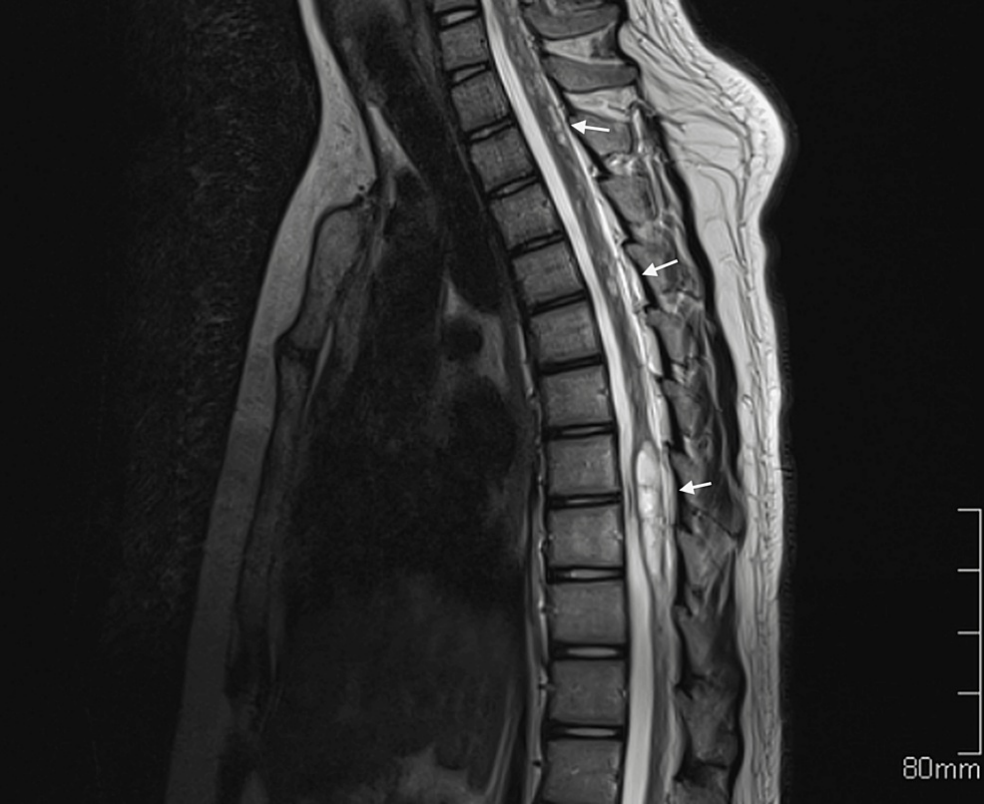
Sagittal T2-weighted spinal imaging showing multiple cystic lesions of varying sizes (arrows)

Further imaging was conducted on the spine, showing multiple hyperintense lesions in T2WI, demonstrating the spread of cysts to the spinal cord (Figure 2).

**Figure 3:**
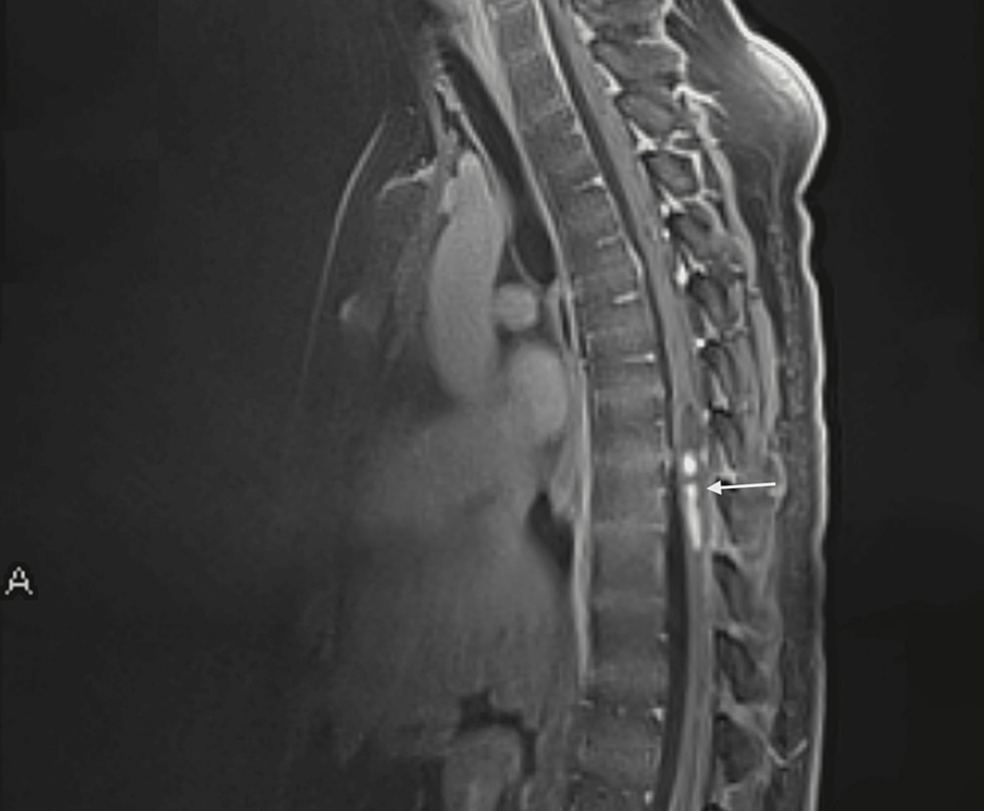
Sagittal spinal contrast-enhanced T1-weighted imaging demonstrating hyperintense enhancing lesions with some cystic segments (arrow), expanding the spinal cord, most prominently around T8-T9

In contrast-enhanced T1-weighted imaging (C+T1WI) of the spine, part of these lesions enhances, demonstrating their mixed nature, particularly in the most prominent lesion around T8-T9 (Figure 3).

**Figure 4:**
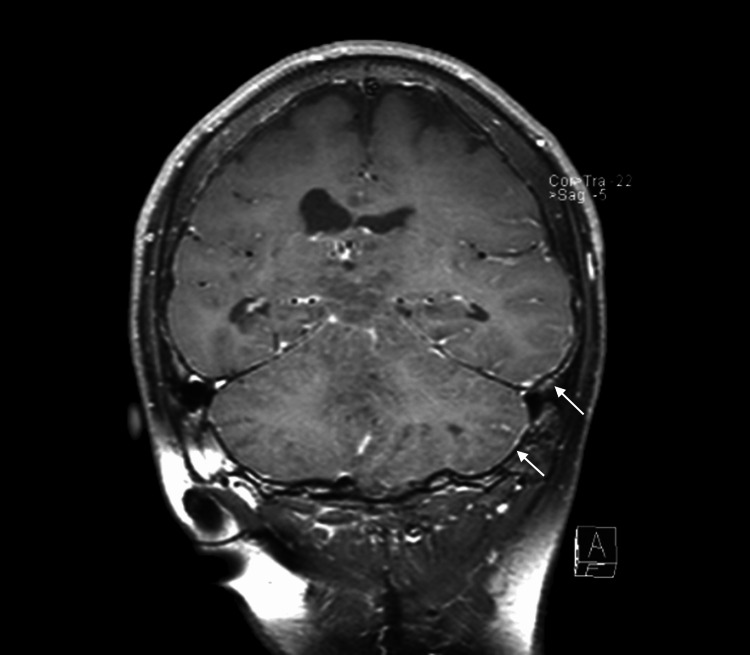
Contrast-enhanced T1-weighted coronal imaging of the brain showing diffuse subpial enhancement (arrows)

C+T1WI of the brain also showed diffuse meningeal enhancement and thickening (Figure 4). The spinal lesions also produced a bulging of the spinal cord and a subsequent localised mass effect. The patient is severely handicapped but currently undergoing treatment.

## Discussion

DL-GNTs are very rare tumours, with fewer than 100 cases reported in the literature since 2012 [3,5]. Of these patients, over two-thirds are reported to survive 10 years or more after diagnosis [6].

The disease clinically presents in a variety of ways including seizures, meningeal signs, neurological deficits, abdominal pain, leg pain, motor delays and signs of increased intracranial pressure such as nausea, headache, abducens palsy and papilledema [2,4]. Case reports of patients initially presenting with neurological symptoms similar to those of our patient, such as facial paresis with cerebellar involvement, exist [3].

Although the imaging presentation of DL-GNT is not fully understood, some features recur in the literature [1]. The first is T2WI hyperintense cystic leptomeningeal-subpial nodular lesions in the posterior fossa [1,3,4]. The second is lesion extension into the spinal cord, revealing a mixed nature when T2WI is compared to C+T1WI, with some nodular enhancement observed [1,4]. A third recurring feature is a leptomeningeal enhancement on C+T1WI [1,3]. Our patient clearly demonstrates all these features. Although imaging features may suggest the diagnosis, a biopsy should be performed for confirmation [1].

Histologically, these tumours appear to have low to moderate density, surrounded by collagen, with cells having an aspect similar to oligodendrogliomas [5]. A small proportion of these cells may feature anaplasia, increased proliferation indices and glomeruloid microvascular changes [5].

Genetically, DL-GNT is often associated with genetic alteration, with 1p deletions in 75% of subjects, BRAF-KIAA fusion in around 55% and BRAF p.V600E substitution mutations in 7% [7]. Two clusters of methylation patterns were discovered in one study, showing 1p deletion either accompanied by 19q deletion or 1q gain [7]. Some evidence exists linking 1p deletion and DL-GNT suggesting a role in favouring dissemination [7]. In our case, a KIAA1549-BRAF was found which, although also associated with pilocytic astrocytomas, is known to be found in DL-GNTs [8].

The optimal treatment of this disease is poorly established owing once more to its rarity [5]. The current treatments include watchful waiting, craniospinal irradiation and, as is strongly suggested by some authors, chemotherapy [5,6].

The differential diagnosis for this entity is vast, mainly owing to the leptomeningeal enhancement, which is non-specific [3,5]. The primary differential diagnosis is bacterial and viral causes, which result in leptomeningeal enhancement [3,5]. Of note is the fact that DL-GNTs are often mistaken for tuberculosis due to their similar appearance, as was the case initially in our patient [3]. Other mimics include central nervous system tumours such as embryonal tumours, germinomas, ependymomas, pineoblastomas and medulloblastomas [3]. For this reason, clinical correlation with the state of the patient is essential in order to narrow down the differential diagnosis [3].

## Conclusions

DL-GNT is a rare and incompletely understood disease. A full radiological description does not yet exist, but some features such as nodules, characteristic extension patterns and post-contrast leptomeningeal enhancement are often found. Current treatments cover a wide range of possibilities, with the optimal treatment for the tumour not yet established. In terms of survival, some authors claim that two-thirds of DL-GNT patients survive over 10 years after the initial diagnosis is made. The imaging differential diagnosis is vast, covering both infectious and tumoural causes, making clinical correlation essential. This case contributes a further example to aid in the establishment of solid radiological criteria and serves to increase awareness of the disease in order to facilitate the diagnosis of future cases.
